# Quantitative Evaluation of Enamel Thickness in Maxillary Central Incisors in Different Age Groups Utilizing Cone Beam Computed Tomography a Retrospective Analysis

**DOI:** 10.3390/diagnostics14222518

**Published:** 2024-11-11

**Authors:** Kinga Mária Jánosi, Diana Cerghizan, Izabella Éva Mureșan, Alpár Kovács, Andrea Szász, Emese Rita Markovics, Krisztina Ildikó Mártha, Silvia Izabella Pop

**Affiliations:** 1 Faculty of Dental Medicine, George Emil Palade University of Medicine, Pharmacy, Science and Technology of Târgu Mureș, 38 Gh. Marinescu Str., 540139 Târgu Mureș, Romania; kinga.janosi@umfst.ro (K.M.J.); izabella-eva.muresan@umfst.ro (I.É.M.); emese.markovics@umfst.ro (E.R.M.); krisztina.martha@umfst.ro (K.I.M.);; 2 Private Practice, 540501 Târgu Mureș, Romania; alparko@yahoo.com (A.K.); andiiszasz@gmail.com (A.S.)

**Keywords:** cone beam computed tomography (CBCT), dental enamel, dental imaging, enamel thickness, prosthodontics

## Abstract

**Background/Objectives:** The presence of enamel on the tooth surface is crucial for the long-term success of minimally invasive adhesive restorations such as dental veneers. Our study aims to evaluate the enamel thickness in the incisal, middle, and cervical portions of the labial surface of the upper central incisors using cone beam computed tomography (CBCT). This imaging method provides detailed and accurate three-dimensional images with a low radiation dose, allowing an accurate assessment of enamel thickness. The analysis aims to identify variations in enamel thickness depending on the age and different levels of the labial tooth surface. **Methods:** 800 CBCT scans performed for diagnostic or therapeutic purposes on patients aged 18–60 years were analyzed. The data were gathered from the imaging archives of private practitioners from Targu Mures and the “George Emil Palade” University of Medicine, Pharmacy, Science, and Technology of Targu Mures. Enamel thickness measurements were conducted using the OnDemand3D Communicator CBCT evaluation program, with subsequent statistical analysis performed using GraphPad Instat Prism software. **Results:** Results showed significant variation in enamel thickness between the incisal, middle, and cervical segments of the labial surface of the upper central incisors. A decrease in enamel thickness with age has been observed. In patients aged 18–40, mean values of enamel thickness 1 mm and 3 mm above the cementoenamel junction (CEJ) were 0.48 ± 0.092, respectively, 0.819 ± 0.158. In patients over 40, the mean values were 0.454 ± 0.116 and 0.751 ± 0.067 at 1 mm, respectively, 3 mm above the CEJ. Statistically significant differences were found between the two age groups at 1 mm and 3 mm above the CEJ, with *p* < 0.0001 and *p* = 0.0214. **Conclusions:** A statistically significant decrease can be observed in enamel thickness in almost the entire labial surface of the upper central incisors with aging. The varied thickness of the enamel at different tooth levels requires individualized planning for each patient to maximize the long-term aesthetic and functional results.

## 1. Introduction

Society considers a perfect smile a sign of beauty, health, and success. Nowadays, patients desire to improve phonetic and masticatory functions and achieve “perfect” aesthetics. Physical appearance has become crucial in defining a person’s identity, and a beautiful smile is essential [1,2]. Modern dentistry is characterized by using minimally invasive tooth preparation with maximum dental hard tissue preservation [3]. The presence of enamel plays an essential role in the long-term success of minimally invasive prosthetic restorations such as dental veneers, ensuring high-quality adhesive cementation [4,5], restoration durability, and accurate tooth shade [6,7,8]. Laminate veneers are commonly used to restore aesthetics and function [9], especially in the frontal area of the dental arches [10,11].

Any deviation from the prescribed protocol could lead to failure and compromise the integrity of the restorations [12,13].

Precisely measuring enamel thickness is essential in avoiding treatment setbacks before tooth preparation or orthodontic stripping procedures.

In a research study focused on porcelain laminate veneer preparation, investigators analyzed the enamel thickness in different sections of the labial surface of maxillary central and lateral incisors. The study demonstrated a significant variation in enamel thickness across different tooth surface regions, with the labial gingival third being the most vital area. These findings highlight the essentiality of careful enamel preservation during tooth preparation for adhesive restorations, especially laminate veneers [14].

Numerous techniques are utilized to measure enamel thickness accurately. A range of traditional and digital tools and techniques are employed for this purpose. Conventional techniques include physical sections and radiographic methods, while digital radiographs, computer-generated micro-CT sections [15,16], Optical Coherence Tomography (OCT) [17], and cone beam computed tomography (CBCT) [18] are examples of digital techniques. These tools and techniques can measure enamel thickness with high precision [19,20,21].

Grine et al. found that measuring enamel thickness using the lateral flat plane radiograph method had limitations [22]. Other studies have suggested an alternative, non-invasive, and potentially reliable method for measuring enamel thickness using CBCT. CBCT can offer a significant advantage over conventional radiography because it provides detailed and accurate three-dimensional (3D) images of dental structures. However, more research is needed to determine the level of precision and reliability of this method [23,24,25]. This technology is commonly used in dentistry, providing high-quality 3D images of anatomical structures with a relatively low radiation dose. It is useful in dental and maxillofacial imaging, allowing clinicians to visualize the teeth, jaws, and surrounding structures in a non-invasive manner. The cone-shaped X-ray beam allows for a more focused and precise image than traditional CT scans, making it a valuable tool for accurate diagnosis and treatment planning [26,27]. It uses multiplanar images, so it is possible to magnify the image, fix certain landmarks, and measure distances between specific anatomical structures. The user-friendly interface of the OnDemand3D software is popular among dental professionals. It is an advanced tool for processing, analyzing, and visualizing 3D images, performing cephalometric analysis, dental measurements, and assessment of facial structures to aid diagnosis and treatment planning. Quality assurance features (tools for image calibration, artifact detection, and image standardization) can help clinicians obtain high-quality diagnostic information. The Dental Volume Reformat—Dental Volume Reformatter (DVR) is the main module of the OnDemand 3D App that provides various formats of 3D images such as axial, panoramic, sagittal section, TMJ, and others [28].

The benefits of CBCT include its ability to produce more explicit images even in the presence of metal restorations or implants, fast processing and viewing of images, and significantly lower radiation doses (up to 96% lower) compared to conventional CT scans. CBCT produces sections of 0.1 mm, whereas CT produces sections with a thickness of 1 mm [29]. Additionally, CBCT is safe for repeated imaging [27] and reliable for measuring accurately the enamel thickness on different tooth surfaces, providing detailed and accurate images that different authors demonstrated in their studies [30,31,32]. Several studies have evaluated the enamel thickness on different portions of the vestibular surface of the maxillary incisors, concluding that there are variations in thickness at different levels related to the patient’s age. However, few studies utilize CBCT examinations for this purpose.

This study aimed to assess the enamel thickness at various segments of the labial surface of the maxillary central incisors and explore potential associations with the age of participants using cone beam computed tomography (CBCT) scans. The null hypothesis was that there is no statistically significant difference in enamel thickness at the entire labial surface of the maxillary central incisors with aging.

## 2. Materials and Methods

### 2.1. Study Design

This retrospective study was conducted at the Faculty of Dental Medicine of the University of Medicine, Pharmacy, Science, and Technology “George Emil Palade” from Targu Mures. This study was designed to evaluate the enamel thickness of patients who had undergone diagnostic or therapeutic procedures at the university and at private dental offices in Targu Mures. This study was carried out in compliance with the Declaration of Helsinki and was approved by the Ethics Committee of the University (3084/22.04.2024). All participants provided written informed consent.

Following the anonymization of data, a single individual with expertise in dental radiology examined standardized CBCT scans of patients aged between 18 and 60. The data were obtained from the university’s imaging archive as well as from various dentists in Targu Mures. The examined records were taken as part of the patients’ diagnostic examinations or for therapeutic purposes, and as such, the patients were not unnecessarily exposed to additional radiation.

### 2.2. Sample Size

The sample size for this study was determined using the G*Power version 3.1.9.6. software (Franz Faul, Universität Kiel, Kiel, Germany) based on a pilot study performed prior. The calculations indicated that a minimum of 359 CBCT scans for each study group (total sample size of 718) would be necessary; this size would provide greater than 95% power to detect significant differences, with an effect size of 0.80 at a significance level of α = 0.05. Thus, 800 CBCT full arch scans were included in this study according to the inclusion and exclusion criteria (Table 1), resulting in a total of 1600 examined maxillary central incisors. Two same-size groups were formed according to age: patients aged 18–40 years and those aged over 40 years.

### 2.3. Imaging Methods Used

During this study, cone beam computed tomography (CBCT) was used as the imaging method. The CBCT scans were taken using a KaVo OP 3D imaging system (KaVo Ltd., Charlotte, NC, USA). The following scanning parameters were used for optimal image quality: 6 milliamperes of tube current (mA), 90 kilovolts (kV) of tube voltage, and a field of view (FOV) of 8 × 15 cm. The scans had a resolution set to a voxel size of 200 μm, considered a standard resolution allowing detailed examination of the anatomical structures. Each patient should be seated with teeth in maximum intercuspation position during the scanning process. The Frankfurt plane must align parallel to the floor; the mid-sagittal plane must remain perpendicular to the ground for accurate and consistent imaging.

### 2.4. Data Collection

The measurements were taken using OnDemand3D Communicator specialized software, version 1.0 (Cybermed, Daejeon, Republic of Korea) using the Dental module.

**Figure 1:**
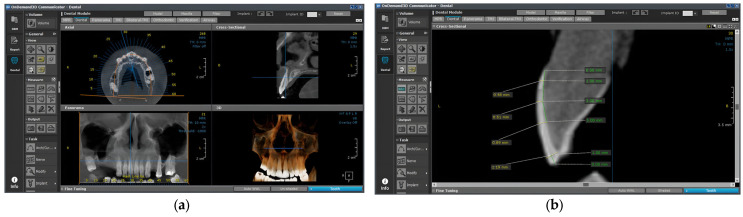
The visualization of the CBCT images: (**a**) the Dental module of the OnDemand3D communicator software version 1.0 (Cybermed, Daejeon, Republic of Korea); (**b**) the landmarks used for the measurements.

The enamel thickness of 1600 maxillary central incisors was recorded in the sagittal profile section of the midline. Four landmarks were selected on the median longitudinal axis of the tooth: (a) 1 mm incisally from cemento-enamel junction (CEJ); (b) 3 mm incisally from CEJ; (c) 5 mm incisally from CEJ; (d) 1 mm apically from the incisal edge (IE). (Figure 1). The tools used (“Taper, Ruler”) were selected from the “Measure” menu.

The enamel thickness was measured by the same operator three times at each landmark. The arithmetic mean of these measurements was obtained, thus determining the result of the measurements. The data obtained were recorded in a database for later analysis.

The standard values were established based on the measurements taken by several authors on the labial surface of the central incisor:

- 1 mm above the cemento–enamel junction (CEJ), enamel thickness ranges from 0.17 mm to 0.52 mm, with a mean thickness of 0.31 mm.
- on the middle third of the surface, 5 mm from the CEJ, ranges from 0.45 mm to 0.93 mm, with a mean thickness of 0.75 mm [19,33,34].

### 2.5. Test–Retest Reliability

The interclass correlation coefficient (ICC) was calculated to determine the reliability of the measurements. The results showed an ICC over 0.75 for all the measurements performed, representing good to excellent reliability. The analysis of the standard error of the measurement showed a variability between 0.023 and 0.035, which demonstrates minimal variation between the measurements.

### 2.6. Statistical Analysis

The resulting data were analyzed using the GraphPad Prism 8 program for macOS (version 10.3.1 (464)). The mean (M), median (Me), and standard deviation (SD) were calculated. The statistical significance was set at *p* < 0.05. Given the non-normal distribution of the data, as confirmed using the Shapiro–Wilk test and the heterogeneity of variances indicated using Levene’s test, we employed the Mann–Whitney test to explore the differences between the enamel thickness related to age and gender. This choice was guided by the test’s suitability for non-parametric data. The Wilcoxon test explored the differences between the values obtained from the performed measurements and the standard values.

### 2.7. Abbreviations

- For the group aged between 18 and 40 years:

11g: right central incisor, 1 mm incisally from CEJ
11c: right central incisor, 3 mm incisally from CEJ
11m: right central incisor, 5 mm incisally from CEJ
11i: right central incisor, 1 mm apically from the IE
21g: left central incisor, 1 mm incisally from CEJ
21c: left central incisor, 3 mm incisally from CEJ
21m: left central incisor, 5 mm incisally from CEJ
21i: left central incisor, 1 mm apically from the IE

- For the group aged over 40 years:

11G: right central incisor, 1 mm incisally from CEJ
11C: right central incisor, 3 mm incisally from CEJ
11M: right central incisor, 5 mm incisally from CEJ
11I: right central incisor, 1 mm apically from the IE
21G: left central incisor, 1 mm incisally from CEJ
21C: left central incisor, 3 mm incisally from CEJ
21M: left central incisor, 5 mm incisally from CEJ
21I: left central incisor, 1 mm apically from the IE

## 3. Results

The mean age of the patients was 41.86 (SD = 11.63), 32.43 (SD = 7.892) for the 18–40 years group, respectively, and 51.30 (SD = 5.360) for patients over 40 years.

The results of the descriptive analysis conducted on the values obtained in the two groups included in the study are presented in Table 2 and Table 3.

**Figure 2:**
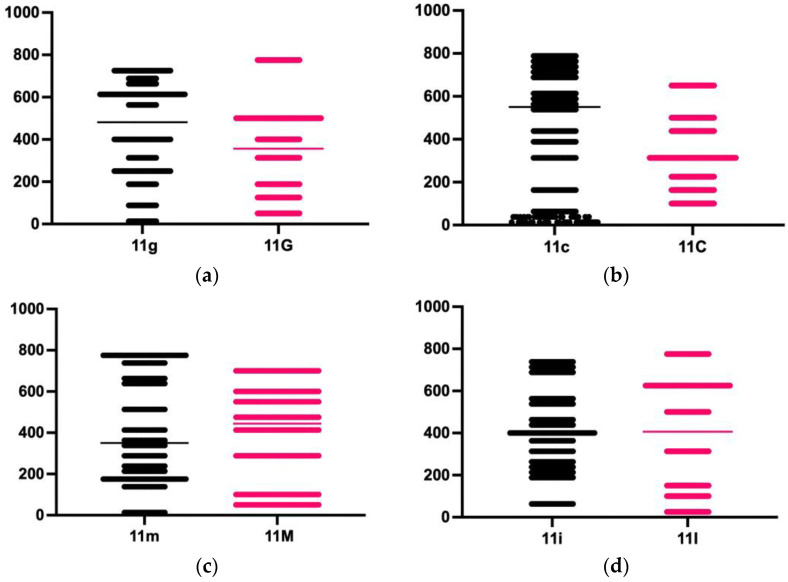
The differences between the values recorded by measuring the thickness of the enamel at the level of the right central incisor in the two groups studied: (**a**) difference at the landmark placed 1 mm incisally from CEJ; (**b**) difference at the landmark placed 3 mm incisally from CEJ; (**c**) difference at the landmark placed 5 mm incisally from CEJ; (**d**) difference at the landmark placed 1 mm apically from the IE.

By comparing the values measured at the level of the right central incisor within the two groups included in the study, using the Mann–Whitney test, no statistically significant differences were found at the landmarks placed 1 mm apically from the IE and at 5 mm incisally from CEJ (Table 4, Figure 2).

**Figure 3:**
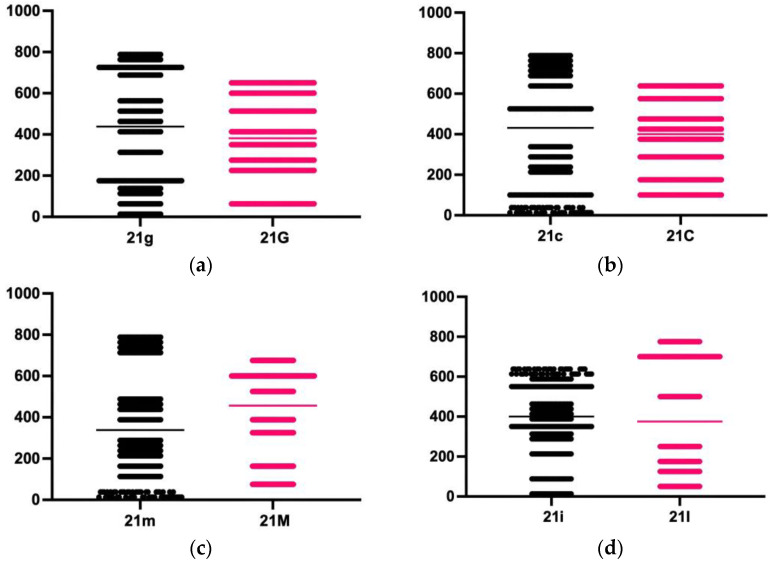
The differences between the values recorded by measuring the thickness of the enamel at the level of the left central incisor in the two groups studied: (**a**) difference at the landmark placed 1 mm incisally from CEJ; (**b**) difference at the landmark placed 3 mm incisally from CEJ; (**c**) difference at the landmark placed 5 mm incisally from CEJ; (**d**) difference at the landmark placed 1 mm apically from the IE.

At the level of the left central incisor, by comparing the measurements made at the selected landmarks within the two groups included in the study, it was found that there is no statistical difference at the landmark positioned 1 mm incisal from the CEJ and that located 1 mm apically from the IE (Table 5, Figure 3).

The values obtained in our study were compared with the mean values from the literature as well as with the maximum values that we considered as standard values. The results are presented in Table 6, Table 7, Table 8 and Table 9.

## 4. Discussion

This study evaluates the enamel thickness at various levels of the labial surface of the upper incisors and explores potential associations with tooth age using CBCT scans.

Several authors have explored enamel thickness using different measurement methods. Ferrari et al. utilized a laboratory caliper with a millimeter scale to measure the enamel thickness of ten maxillary anterior teeth designated for ceramic veneers without considering the patient’s age. Different enamel thicknesses were recorded at different levels of the labial surface: 0.4 mm gingivally, 0.9 mm in the middle third, and 1.0 mm at the incisal third [35]. Others [19,36,37] evaluated the labial enamel thickness of upper incisors by scanning electron microscope (SEM) or CBCT at 1, 3, and 5 mm distances from the CEJ to analyze the correlation between chronological age and enamel thickness in a population aged between 35 and 70 years, with 0.28 mm, 0.50 mm, and 0.73 mm thicknesses, and an inverse correlation between age and enamel thickness (1 mm = 0.31 ± 0.01; 3 mm = 0.54 ± 0.01; 5 mm = 0.75 ± 0.02/0.4 mm, 0.6 mm, 0.9 mm average values). Huysmans et al. proved the applicability of ultrasonic measurements for determining enamel thickness. At the same time, Louwerse pointed out the method’s limitations, such as the inability to detect thickness changes of less than 0.33 mm [38,39]. Smith et al. used an invasive method to examine enamel thickness on the bucolingual cross-section of extracted molars on micrographs related to population and sex [40]. There have also been reported attempts to determine enamel thickness using lateral radiographs with a parallel film technique. The measurements obtained on the radiographs were compared with those from the longitudinal cross-section of the molars. This method has proven unprecise [22]. In other studies, without sex and age references, micro-CT or periapical radiographs were used to determine the enamel thickness on maxillary premolars and canines, proving the reliability and high accuracy of the method [32,41].

We can find similar studies about enamel thickness evaluation based on CBCT measurements in the literature. Brokos et al. demonstrated by CBCT measurements that the enamel thickness of the upper incisors decreases with age. The examined teeth were divided into three groups according to age. The obtained average values were young (846 µm), middle (758 µm), and aged (705 µm), with higher values in females. The location of the teeth did not influence the values; central and lateral incisors showed similar mean values [25].

The statistical analysis of our values obtained after the measurements showed no differences between the two age groups studied in most enamel thickness landmarks. Small but statistically significant differences were observed 1 mm above the CEJ. A similar study by Kunin et al. [42] revealed reduced enamel thickness in older people, especially in the gingival area. By comparing the values obtained through the measurements with the standard values selected from several studies, it was demonstrated that the values obtained in this study are lower, especially in the group over 40 years old. Our mean values recorded at the level of the incisal area and the middle third of the labial surface, regardless of age, are higher than those at the cervical level. Our findings follow those obtained by Mohamed, who concluded that dentin exposure must be avoided during tooth preparation, critically at the cervical level [6]. The reduced enamel thickness at this level, especially in older people, as observed in our study, requires rigorous treatment planning with the help of minimally invasive restorations to avoid compromising them. The same results were reported in a study conducted by Pahlevan et al., who performed measurements after tooth preparation for veneers and emphasized the importance of hard dental tissue preservation, especially at the cervical level [14]. The central incisors on both sides of the dental arch exhibit approximately equal enamel thickness, similar within each age category. By understanding these mean values, practitioners with varying experience levels can easily apply a standardized approach to minimally invasive preparation. Considering these values obtained using depth guidance techniques, primarily through mock-up preparation, excessive preparation, and the occurrence of dentin islands, which could compromise the longevity of the restoration, it can be avoided. Combining various magnification techniques with the mock-up preparation technique can significantly reduce excessive tooth structure removal, preserving hard dental tissues. In the case of preparation for veneers, the correct choice of bur size depends on the enamel thickness and the material from which the future restoration will be fabricated.

This study uses CBCT imaging to provide information about enamel thickness variations of upper central incisors. The findings may enhance dental treatment planning and prosthetic interventions in minimally invasive clinical practice. The quantity and quality of the remaining enamel after preparation can significantly influence the durability of the restoration from the cementation process onwards. The thicker the layer of remaining enamel and enamel–ceramic is, the more resistant the veneer is to the forces that can cause its fracture [12]. According to Yāgci et al., obtaining a maximal shear bond strength and optimal marginal sealing is necessary to maintain tooth preparation only in the enamel [43]. The statistical analysis of our values obtained after the measurements showed no differences between the two age groups studied at most enamel thickness landmarks. Small but statistically significant differences were observed 1 mm above the CEJ. A similar study by Kunin et al. [42] revealed reduced enamel thickness in older people, especially in the gingival area.

Limitations of the study: As this was a retrospective study, the factors influencing the quality and uniformity of the data recorded (ex., patient positioning) through CBCT could not be controlled. The measurements were made only at the level of the labial surface of the central incisors. Factors that could influence enamel thickness, such as previous orthodontic treatments, the level of dental wear, and the patients’ sex, were not considered. The study groups were limited to a specific geographical location, which may affect the generalization of the results. These limitations underscore the need for further research to enhance the applicability of the findings, making the research more inclusive and impactful. Future studies should be extended to other dental groups and a more diverse population by considering a broader range of factors influencing enamel thickness, such as diet, dental hygiene, genetic aspects, and patients’ gender, which will lead to a more comprehensive individualization of treatment planning, especially in minimally invasive dentistry.

## 5. Conclusions

Based on the findings of this study, the following conclusions were drawn:

The null hypothesis is rejected. A statistically significant decrease can be observed in enamel thickness in almost the entire labial surface of the upper central incisors with aging. The precise evaluation of enamel thickness is essential during the treatment planning for minimally invasive prosthetic rehabilitation with dental veneers. The enamel thickness variations at different levels of the tooth surface require individualized planning for each patient to maximize the long-term success of aesthetic and functional adhesive restorations.

## Figures and Tables

**Table 1:** Inclusion and exclusion criteria for the CBCT records.

Inclusion Criteria	Exclusion Criteria
Patients over 18 years	Patients younger than 18 years
Permanent dentition	Mixed/deciduous dentition
Presence of both maxillary central incisors	Malaligned maxillary central incisors
Sound permanent maxillary central incisors (no caries, endodontic treatments, or restorations)	Supernumerary teeth
	No shape or structural abnormalities
Completely erupted maxillary central incisors	Orthodontic treatment in progress
Full arch scans	Poor technical quality of the scans

**Table 2:** Descriptive statistics for the group aged between 18 and 40 years.

	Mean (m)	Median (M)	Minimum (min)	Maximum (Max)	Std. Deviation (SD)	Std. Error of Mean	Lower 95% CI of Mean	Upper 95% CI of Mean
11g	0.48	0.48	0.28	0.61	0.092	0.005	0.471	0.489
11c	0.819	0.835	0.49	1.06	0.158	0.008	0.803	0.834
11m	0.964	0.955	0.6	1.19	0.151	0.008	0.95	0.979
11i	1.106	1.1	0.81	1.43	0.146	0.007	1.092	1.121
21g	0.498	0.48	0.28	0.78	0.136	0.007	0.485	0.511
21c	0.806	0.79	0.53	1.08	0.162	0.008	0.79	0.822
21m	0.936	0.925	0.63	1.18	0.143	0.007	0.922	0.95
21i	1.078	1.105	0.73	1.27	0.138	0.007	1.065	1.092

**Table 3:** Descriptive statistics for the group aged over 40 years.

	Mean (m)	Median (M)	Minimum (min)	Maximum (Max)	Std. Deviation (SD)	Std. Error of Mean	Lower 95% CI of Mean	Upper 95% CI of Mean
11G	0.454	0.455	0.3	0.72	0.116	0.006	0.442	0.465
11C	0.751	0.73	0.67	0.9	0.067	0.003	0.745	0.758
11M	0.959	1.01	0.75	1.1	0.13	0.007	0.946	0.972
11I	1.095	1.11	0.79	1.57	0.232	0.012	1.072	1.118
21G	0.46	0.46	0.36	0.55	0.059	0.003	0.454	0.466
21C	0.776	0.795	0.65	0.89	0.081	0.004	0.768	0.784
21M	0.964	0.975	0.8	1.09	0.097	0.005	0.954	0.973
21I	1.09	1.085	0.77	1.35	0.21	0.01	1.069	1.111

**Table 4:** Mann–Whitney test results for the median values of the right central incisors related to the different age groups.

Selected Landmarks	Difference Between Medians	*p*-Value
1mm incisally from CEJ	0.0250	<0.0001
1 mm apically from the IE	0.010	0.1797
5 mm incisally from CEJ	0.055	0.7017
3 mm incisally from CEJ	−0.105	<0.0001

**Table 5:** Mann–Whitney test results for the median values of the left central incisors, related to the different age groups.

Selected Landmarks	Difference Between Medians	*p*-Value
1mm incisally from CEJ	−0.020	0.0845
1 mm apically from the IE	0.005	0.7739
3 mm incisally from CEJ	0.050	0.0214
5 mm incisally from CEJ	0.005	0.0214

**Table 6:** The discrepancies recorded between the values measured at 1 mm above the CEJ obtained within the study groups and the mean of the standard values.

	Discrepancy	Mean of the Standard Values	*p*-Value
11g	0.170	0.31	<0.0001
11G	0.145		
21g	0.170		
21G	0.150		

**Table 7:** The discrepancies recorded between the values measured at 1 mm above the CEJ obtained within the study groups and the maximum standard values.

	Discrepancy	Mean of the Standard Values	*p*-Value
11g	−0.04	0.52	<0.0001
11G	−0.065		
21g	−0.04		
21G	−0.060		

**Table 8:** The discrepancies recorded between the values measured at 5 mm incisally from CEJ obtained within the study groups and the mean of the standard values.

	Discrepancy	Mean of the Standard Values	*p*-Value
11m	0.205	0.75	<0.0001
11M	0.260		
21m	0.175		
21M	0.225		

**Table 9:** The discrepancies recorded between the values measured at 5 mm incisally from CEJ obtained within the study groups and the maximum standard values.

	Discrepancy	Mean of the Standard Values	*p*-Value
11m	0.025	0.93	<0.0001
11M	0.080		0.0293
21m	−0.005		0.9998
21M	0.045		<0.0001

## Data Availability

The datasets analyzed during this study are available from the first author on request.

## References

[1] AlSagob E.I., Alkeait F., Alhaimy L., Alqahtani M., Hebbal M., Ben Gassem A.A. (2021). Impact of Self-Perceived Dental Esthetic on Psycho-Social Well-Being and Dental Self Confidence: A Cross-Sectional Study Among Female Students in Riyadh City. Patient Prefer. Adherence.

[2] Aishwarya A., Aiswarya D.S., Sasi A.I., Akshaya V.S., Subodh A., Praveen D. (2023). Dental Aesthetics and Its Impact on Psychosocial Wellbeing among Students of Dental Colleges in South Kerala. Int. J. Dent. Med. Sci. Res..

[3] Yu H., Zhao Y., Li J., Luo T., Gao J., Liu H., Liu W., Liu F., Zhao K., Liu F. (2019). Minimal Invasive Microscopic Tooth Preparation in Aesthetic Restoration: A Specialist Consensus. J. Esthet. Restor. Dent..

[4] Aminian A., Brunton P.A. (2003). A Comparison of the Depths Produced Using Three Different Tooth Preparation Techniques. J. Prosthet. Dent..

[5] Öztürk E., Bolay Ş., Hickel R., Ilie N. (2013). Shear Bond Strength of Porcelain Laminate Veneers to Enamel, Dentine and Enamel–Dentine Complex Bonded with Different Adhesive Luting Systems. J. Dent..

[6] Mohamed M. (2022). Effect of Preparation Depth on the Fracture Resistance of Two Monolithic Ceramic Laminate Veneers. Egypt. Dent. J..

[7] Zhu J., Gao J., Jia L., Tan X., Xie C., Yu H. (2022). Shear bond strength of ceramic laminate veneers to finishing surfaces with different percentages of preserved enamel under a digital guided method. BMC Oral Health.

[8] Alavi A.A., Behroozi Z., Nik Eghbal F. (2017). The shear bond strength of porcelain laminate to prepared and unprepared anterior teeth. J. Dent..

[9] Vanlıoğlu B.A., Kulak-Özkan Y. (2014). Minimally invasive veneers: Current state of the art. Clin. Cosmet. Investig. Dent..

[10] Cherukara G., Seymour K., Samarawickrama D. (2002). A Study into the Variations in the Labial Reduction of Teeth Prepared to Receive Porcelain Veneers—A Comparison of Three Clinical Techniques. Br. Dent. J..

[11] Cherukara G.P., Seymour K.G., Zou D.Y.D., Samarawickrama D.Y.D. (2003). Geographic Distribution of Porcelain Veneer Preparation Depth with Various Clinical Techniques. J. Prosthet. Dent..

[12] Ge C., Green C.C., Sederstrom D., McLaren E.A., White S.N. (2014). Effect of porcelain and enamel thickness on porcelain veneer failure loads in vitro. J. Prosthet. Dent..

[13] Burke F.T. (2012). Survival rates for porcelain laminate veneers with special reference to the effect of preparation in dentin: A literature review. J. Esthet. Restor. Dent..

[14] Pahlevan A., Mirzaee M., Yassine E., Ranjbar Omrany L., Hasani Tabatabaee M., Kermanshah H., Arami S., Abbasi M. (2014). Enamel Thickness After Preparation of Tooth for Porcelain Laminate. J. Dent..

[15] Gaboutchian A.V., Knyaz V.A., Maschenko E.N., Dac L.X., Maksimov A.A., Emelyanov A.V., Korost D.V., Stepanov N.V. (2023). Measuring Dental Enamel Thickness: Morphological and Functional Relevance of Topographic Mapping. J. Imaging.

[16] Bijle M.N., Mallineni S.K., Tsoi J. (2022). Qualitative and Quantitative Micro-CT Analysis of Natal and Neonatal Teeth. Children.

[17] Miyagi H., Oki K., Tsukiyama Y., Ayukawa Y., Koyano K. (2022). Assessment of the Accuracy in Measuring the Enamel Thickness of Maxillary Incisors with Optical Coherence Tomography. Diagnostics.

[18] Hakami Z., Marghalani H.Y., Hedad I., Khawaji M., Abutaleb G., Hakami A., Almoammar S., Alshehri A. (2023). Comparison of Tooth Color and Enamel and Dentinal Thickness between Orthodontically Treated and Untreated Individuals. Diagnostics.

[19] Atsu S.S., Aka P.S., Kucukesmen H.C., Kilicarslan M.A., Atakan C. (2005). Age-Related Changes in Tooth Enamel as Measured by Electron Microscopy: Implications for Porcelain Laminate Veneers. J. Prosthet. Dent..

[20] Trivedi A., Trivedi S., Chhabra S., Bansal A., Jain A., Kaushal P., Sachdeva S., Kukreja N. (2022). “It doesn’t matter what lost what matter is what remains” R.D.T (Remaining Dentin Thickness): A review. J. Pharm. Negat. Results.

[21] Olejniczak A.J., Grine F.E. (2006). Assessment of the accuracy of dental enamel thickness measurements using microfocal X-ray computed tomography. Anat. Rec. A Discov. Mol. Cell Evol. Biol..

[22] Grine F.E., Stevens N.J., Jungers W.L. (2001). An evaluation of dental radiograph accuracy in the measurement of enamel thickness. Arch. Oral Biol..

[23] Plotino G., Grande N.M., Pecci R., Bedini R., Pameijer C.H., Somma F. (2006). Three-dimensional imaging using microcomputed tomography for studying tooth macromorphology. J. Am. Dent. Assoc..

[24] Baumgaertel S., Palomo J.M., Palomo L., Hans M.G. (2009). Reliability and accuracy of cone-beam computed tomography dental measurements. Am. J. Orthod. Dentofac. Orthop..

[25] Brokos Y.P., Stavridakis M., Bortolotto T., Krejci I. (2015). Evaluation of enamel thickness of upper anterior teeth in different age groups by Dental Cone Beam Computed Tomography Scan, in vivo. Int. J. Adv. Case Rep..

[26] Pop S.I., Cerghizan D., Mițariu L., Jánosi K.M., D’Andrea A. (2024). CBCT Evaluation of Alveolar Bone Change and Root Resorption after Orthodontic Treatment: A Retrospective Study. Diagnostics.

[27] Issrani R., Issrani R., Ganji K. (2022). Cone-Beam Computed Tomography: A New Tool on the Horizon for Forensic Dentistry. Int. J. Environ. Res. Public Health.

[28] Băciuţ M.F. (2007). Implantologie Orală.

[29] Khalil S.K., Mudhir A.M., Sirri M.R. (2023). Accuracy of CBCT and Intraoral Scanner Images for Measuring Tooth Widths and Bolton’s Ratio: A Comparative Study with Gold Standard (Plaster Models) in Duhok’s Adult Population. SN Appl. Sci..

[30] Wang Y., He S., Yu L., Li J., Chen S. (2011). Accuracy of Volumetric Measurement of Teeth in Vivo Based on Cone Beam Computer Tomography. Orthod. Craniofac. Res..

[31] Abulhamael A.M., Barayan M., Makki L.M., Alsharyoufi S.M., Albalawi T.H.S., Zahran S., Alkhattab O., Kutbi A.S., Alrehili R.S., Alzamzami Z.T. (2024). The Accuracy of Cone Beam Computed Tomography Scans in Determining the Working Length in Teeth Requiring Non-surgical Endodontic Treatment: A Retrospective Clinical Study. Cureus.

[32] Aktuna Belgın C., Serindere G., Orhan K. (2019). Accuracy and Reliability of Enamel and Dentin Thickness Measurements on Micro-Computed Tomography and Digital Periapical Radiographs. J. Forensic Radiol. Imaging.

[33] Jacobson N., Frank C.A. (2008). The Myth of Instant Orthodontics: An Ethical Quandary. J. Am. Dent. Assoc..

[34] Shillingburg H.T., Grace C.S. (1973). Thickness of Enamel and Dentin. J. South Calif. Dent. Assoc..

[35] Ferrari M., Patroni S., Balleri P. (1992). Measurement of Enamel Thickness in Relation to Reduction for Etched Laminate Veneers. Int. J. Periodontics Restor. Dent..

[36] Kedici P.S., Atsü S., Gökdemir K., Sarikaya Y., Gürbüz F. (2000). Micrometric Measurements by Scanning Electron Microscope (SEM) for Dental Age Estimation in Adults. J. Forensic Odontostomatol..

[37] Al-Zahawi A.R., Ibrahim R.O., Talabani R.M., Dawood S.N., Garib DS H., Abdalla A.O. (2023). Age and Sex Related Change in Tooth Enamel Thickness of Maxillary Incisors Measured by Cone Beam Computed Tomography. BMC Oral Health.

[38] Huysmans M.C.D.N.J.M., Thijssen J.M. (2000). Ultrasonic Measurement of Enamel Thickness: A Tool for Monitoring Dental Erosion?. J. Dent..

[39] Louwerse C., Kjaeldgaard M., Huysmans M.C.D.N.J.M. (2004). The Reproducibility of Ultrasonic Enamel Thickness Measurements: An In Vitro Study. J. Dent..

[40] Smith T.M., Olejniczak A.J., Reid D.J., Ferrell R.J., Hublin J.J. (2006). Modern Human Molar End Thickness and Enamel–Dentine Junction Shape. Arch. Oral Biol..

[41] Akli E., Araujo E.A., Kim K.B., McCray J.F., Hudson M.J. (2020). Enamel Thickness of Maxillary Canines Evaluated with Microcomputed Tomography Scans. Am. J. Orthod. Dentofac. Orthop..

[42] Kunin A.A., Evdokimova A.Y., Moiseeva N.S. (2015). Age-Related Differences of Tooth Enamel Morphochemistry in Health and Dental Caries. EPMA J..

[43] Yağci F.İ.L.İ.Z., Türker G., Yılancı H. (2021). Determination of the Thickness of the Safe Enamel for Laminate Veneer Preparation and Orthodontic Stripping by CBCT. Niger. J. Clin. Pract..

